# Understanding the Configurational Entropy Evolution in Metal‐Phosphorus Solid Solution for Highly Reversible Li‐Ion Batteries

**DOI:** 10.1002/advs.202300271

**Published:** 2023-02-15

**Authors:** Yaqing Wei, Runzhe Yao, Xuhao Liu, Wen Chen, Jiayao Qian, Yiyi Yin, De Li, Yong Chen

**Affiliations:** ^1^ State Key Laboratory of Marine Resource Utilization in South China Sea Hainan Provincial Key Laboratory of Research on Utilization of Si‐Zr‐Ti Resources School of Materials Science and Engineering Hainan University 58 Renmin Road Haikou Hainan 570228 P. R. China; ^2^ Guangdong Key Laboratory for Hydrogen Energy Technologies School of Materials Science and Hydrogen Energy Foshan University Foshan 528000 P. R. China

**Keywords:** anode material, high entropy alloy, initial coulombic efficiency, lithium‐ion batteries, volume expansion

## Abstract

The high‐entropy materials (HEM) have attracted increasing attention in catalysis and energy storage due to their large configurational entropy and multiunique properties. However, it is failed in alloying‐type anode due to their Li‐inactive transition‐metal compositions. Herein, inspired by high‐entropy concept, the Li‐active elements instead of transition‐metal ones are introduced for metal‐phosphorus synthesis. Interestingly, a new Zn_x_Ge_y_Cu_z_Si_w_P_2_ solid solution is successfully synthesized as proof of concept, which is first verified to cubic system in F‐43m. More specially, such Zn_x_Ge_y_Cu_z_Si_w_P_2_ possesses wide‐range tunable region from 9911 to 4466, in which the Zn_0.5_Ge_0.5_Cu_0.5_Si_0.5_P_2_ accounts for the highest configurational entropy. When served as anode, Zn_x_Ge_y_Cu_z_Si_w_P_2_ delivers large capacity (>1500 mAh g^−1^) and suitable plateau (≈0.5 V) for energy storage, breaking the conventional view that HEM is helpless for alloying anode due to its transition‐metal compositions. Among them, the Zn_0.5_Ge_0.5_Cu_0.5_Si_0.5_P_2_ exhibits the highest initial coulombic efficiency (ICE) (93%), Li‐diffusivity (1.11 × 10^−10^), lowest volume‐expansion (34.5%), and best rate performances (551 mAh g^−1^ at 6400 mA g^−1^) owing to its largest configurational entropy. Possible mechanism reveals the high entropy stabilization enables good accommodation of volume change and fast electronic transportation, thus supporting superior cyclability and rate performances. This large configurational entropy strategy in metal‐phosphorus solid solution may open new avenues to develop other high‐entropy materials for advanced energy storage.

## Graphical abstract

Inspired by the high entropy concept, a new novel Zn_x_Ge_y_Cu_z_Si_w_P_2_ high entropy material (HEM) is successfully synthesized, which is enable a large capacity over 1500 mAh g^−1^ for LIBs, breaking the conventional view that high entropy alloy is helpless for alloying anode due to its Li‐inactive transition metal compositions. Among them, the Zn_0.5_Ge_0.5_Cu_0.5_Si_0.5_P_2_ exhibits the highest initial coulombic efficiency (ICE = 93%), Li diffusion coefficient (1.11 × 10^−11^), lowest volume expansion (34.5%), and best rate performances (551 mAh g^−1^ at 6400 m g^−1^) owing to its highest configurational entropy.

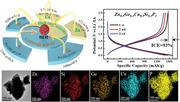

## 1 Introduction

The Li‐ion batteries (LIBs), with great advantages of environmental friendly, high energy conversion efficiency and nonmemory effect, have been widely applied for electric vehicles (EVs) and hybrid electric vehicles (HEVs) market.^[^ ^1^ ^]^ However, recent commercial graphite anode in LIBs, with low capacity of only 372 mAh g^−1^, can't be further enhanced the energy density (>500 Wh kg^−1^) output and failed to meet the driving mileage demand (>800 km) for EVs/HEVs.^[^ ^2^ ^]^ Compared to graphite, the emerging alloying type materials are proposed to be served as the alternative anodes for LIBs due to their much higher capacity than graphite by multielectron reaction.^[^ ^3^ ^]^ In recent decades, various alloying anode materials including the Si (4200 mAh g^−1^),^[^ ^4^ ^]^ Ge (1600 mAh g^−1^),^[^ ^5^ ^]^ Sn (994 mAh g^−1^),^[^ ^6^ ^]^ P (2596 mAh g^−1^),^[^ ^7^ ^]^ Sb (660 mAh g^−1^),^[^ ^8^ ^]^ and Bi (384 mAh g^−1^)^[^ ^9^ ^]^ have been widely investigated and greatly facilitated the capacity output to a large extent. Though quite attractive for those alloying materials, challenge is that such elementary substances still suffer large volume expansion during lithiation (up to 400% for Si), resulting in serious particle pulverization and fast capacity fading.^[^ ^10^ ^]^ Owing to the repetitive volume changes, the lithium and electrolyte are continuously consumed, leading to a low initial coulombic efficiency (ICE < 75%) as well.^[^ ^11^ ^]^ Besides, the electronic conductivity of above semiconducting analogues (2.5 × 10^−4^ S m^−1^ of Si, 1 × 10^−12^ S m^−1^ of P) is too low to support the good reaction kinetics and fast charging performances.^[^ ^12^ ^]^ Even though much effort on morphology modification and nanostructure design (such as nanowire, core–shell structure, and carbon composite) has been made,^[^ ^13^ ^]^ the fast capacity fading, low ICE and unsatisfactory rate performance of above alloying substances can't still be addressed thoroughly.

Recently, the high entropy materials (HEM), as an emerging concept, have attracted increasing attention in the fields of catalysis, thermoelectricity, superionic conductivity, and energy storage.^[^ ^14^ ^]^ Generally, the HEM represents one kind multielement systems crystallizing into a new single phase, in which five or more major elements share the equiatomic sites to stabilize at a solid solution state.^[^ ^15^ ^]^ By reasonably designing elemental components, the compositional flexibility allows the HEM to develop series advanced materials with target properties, which can't be obtained by traditional materials with only one or several dominant elements.^[^ ^16^ ^]^ Benefitted from the large configurational entropy, the HEM shows great advantages and unique properties including the superionic conductivity ((MgCoNiCuZnGaLiO),^[^ ^17^ ^]^ high yield strength (CrFeCoNiPd),^[^ ^18^ ^]^ superior catalytic activity (PtPdRhRuCe),^[^ ^19^ ^]^ and good energy storage potential (Mg_0.2_Mn_0.2_Ni_0.2_Co_0.2_Zn_0.2_Fe_2_O_4_).^[^ ^20^ ^]^ The configurational entropy in battery electrode material, if successfully introduced, would become highly attractive as it promotes the simultaneous optimization of multiple properties.^[^ ^21^ ^]^ More specially, if we extend the above single alloying substances into one kind high entropy alloy system and implement the configurational entropy working in anode material, one would expect that increasing the number of elemental components to larger configurational entropy in HEM would buffer the large volume expansion in a harmonious and gentle way by lithiation at different plateau and preventing the formation of a single dominant lithiated product.^[^ ^22^ ^]^ Besides, the large configurational entropy and high conductivity are believed to promote fast electronic transportation toward better performance as well.^[^ ^23^ ^]^ As proved, the high entropy concept has greatly applied into LIB‐cathode materials (e.g., Li_1.3_Mn^2+^_0.1_Co^2+^_0.1_Mn^3+^_0.1_Cr^3+^_0.1_Ti_0.1_Nb_0.2_O_1.7_F_0.3_,^[^ ^24^ ^]^ Na_3_V_1.9_(CaMgAlCrMn)_0.1_(PO_4_)_2_‐F_3_,^[^ ^25^ ^]^ and NaNi_0.12_Cu_0.12_Mg_0.12_Fe_0.15_Co_0.15_Mn_0.1_Ti_0.1_Sn_0.1_Sb_0.04_O_2_^[^ ^26^ ^]^). However, when applied into anode electrode, all of the reported HEMs are composed of transition metal components (Fe, Ni, Cr, etc.), which are helpless for Li‐storage and can't be applied into the alloying‐type anode for LIBs.^[^ ^27^ ^]^ Up to now, to the best of our knowledge, alloying type HEMs are rarely reported due to their transition metal components and failure of Li‐active property.

Herein, inspired by the high entropy concept and configurational entropy evolution, the HEM anode is rationally designed by introducing the Li‐active elements (Si, Ge, P, and Zn) instead of transition metal components. Interestingly, a new novel HEM composed of Zn_x_Ge_y_Cu_z_Si_w_P_2_ is successfully synthesized as a proof of concept, which is first verified to belong to the cubic system in F‐43m space group, 216. Different from conventional HEMs, such Zn_x_Ge_y_Cu_z_Si_w_P_2_ products enable a large reversible capacity over 1500 mAh g^−1^ for LIBs, breaking the limitation that typical HEM can't be worked in alloying‐type anode due to its Li‐inactive transition metal composition. More specially, those discovered HEMs of cation‐disorder Zn_x_Ge_y_Cu_z_Si_w_P_2_ possess a wide range tunable configurational entropy region (from 9911 to 4466), which is considered as one kind of unique solid solution rather than normal metal phosphides. Among them, the Zn_0.5_Ge_0.5_Cu_0.5_Si_0.5_P_2_ (HEM‐5555) accounts for the highest configurational entropy, mitigating the industry's reliance on any single critical metal source as well. Various experimental tools and theoretical calculation are performed to investigate the structural and physicochemical properties of those HEMs. When served as the anode for LIBs, all of the above HEM analogues deliver large capacity (1500 mAh g^−1^) and suitable plateau (≈0.5 V) for energy storage. Similarly, the Zn_0.5_Ge_0.5_Cu_0.5_Si_0.5_P_2_ exhibits the highest ICE (93%), Li diffusivity (1.01 × 10^−14^), lowest volume expansion (34.5%), and more specially, the best rate performances (551 mAh g^−1^ at 6400 mA g^−1^) owing to its highest configurational entropy. Possible mechanism investigation reveals the high entropy stabilization help to buffer the volume change and facilitate electronic transportation to a large extent, thus supporting the reversibility, cyclability, and better rate performances for LIBs. This new finding of novel Zn_x_Ge_y_Cu_z_Si_w_P_2_ solid solution will inject fresh blood into alloying anode family for fast charging LIBs. And the enlistment of large configurational entropy into alloying anode may provide a new strategy for material design and optimization toward advanced energy storage.

## 2 Result and Discussion

### 2.1 Rational Design of Elemental Compositions for HEM Synthesis

**Figure 1:**
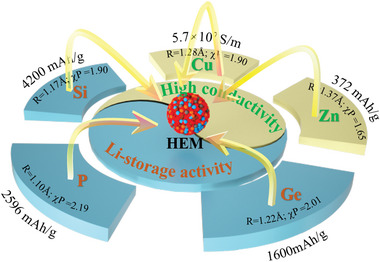
Rational design of elementary selection for the high entropy material (HEM) synthesis.

Generally speaking, traditional HEMs are always synthesized by transition metal elements because of their similar atomic‐radius and electronegativity.^[^ ^28^ ^]^ For the metal or nonmetal elements, however, are more likely to form the typical ionic or covalent compounds rather than solid solution alloys. It is very difficult to obtain one kind HEM that is synthesized by metal and nonmetal elements due to their quite large differences on atomic radius and electronegativity.^[^ ^29^ ^]^ Recently, the reported semimetal Ge and nonmetal P are surprisingly found fused into a high conductive alloy rather than ionic compounds, which give us the light to synthesize HEM using the Li‐active elements with similar atomic radius and electronegativity as starting materials.^[^ ^30^ ^]^ As shown in **Figure** **1**, the typical alloying substances with Li‐active property, including the Si (4200 mAh g^−1^, Li_4.4_Si), Ge (1600 mAh g^−1^, Li_4.4_Ge), Sn (994 mAh g^−1^, Li_4.4_Sn), P (2596 mAh g^−1^, Li_3_P), Sb (660 mAh g^−1^, Li_3_Sb), Bi (384 mAh g^−1^, Li_3_Bi), and Zn (410 mAh g^−1^, LiZn) are taken into account for the HEM synthesis. Further considering the atomic radius and electronegativity of above analogues, the target atoms of Si, Ge, P, and Zn are found possessed the similar radius (*R* = 1.10–1.37 Å) and close electronegativity (*χ*P = 1.65–2.19). According to the Hume–Rothery Rules on radius and electronegativity differences (Δ*R* = 19% < 30%, Δ*χ*P = 25% < 0.4), such congenial analogues are potentially able to crystallize in a single phase, simultaneously sharing the equiatomic sites and configurational entropy. Besides, in addition to Li‐active component, the high conductive elements also need to be contained for the HEM design to keep its high conductivity output. Therefore, the elemental Cu, with great advantages of high conductivity (5.7 × 10^7^ S m^−1^), low price, abundant resource, and more specially, the similar atomic radius (*R* = 1.28 Å) and electronegativity (*χ*P = 1.90) with above Li‐active components, is proposed for the new HEM design as well. Consequently, the well‐designed Li‐active Si, Ge, P, Zn, and high conductive Cu elements are introduced for the HEM design and synthesis.

### 2.2 Synthesis and Characterization of New Zn_x_Ge_y_Cu_z_Si_w_P_2_ Phase

**Figure 2:**
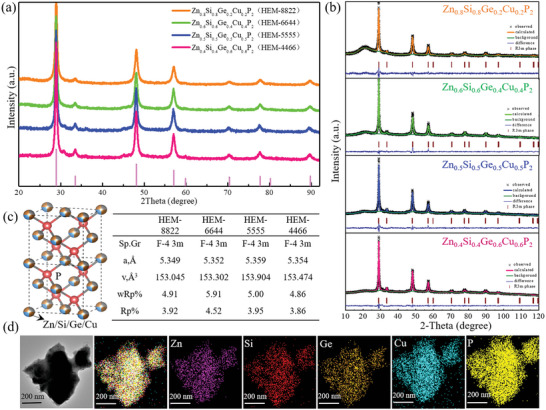
Synthesis of series Zn_x_Ge_y_Cu_z_Si_w_P_2_ solid solutions. The X‐ray diffraction (XRD) patterns in different proportion (a), powder XRD patterns for Rietveld structure analysis (b), crystal structure and Rietveld refinement of cell parameters (c), transmission electron microscopy (TEM) image and elementary mapping (d) for Zn_x_Ge_y_Cu_z_Si_w_P_2_ analogues.

Firstly, the HEM of Zn‐Si‐Cu‐Ge‐P was synthesized by a high energy mechanical ball milling way using its simple substances as starting materials. Interestingly, after fusing together, the formed Zn_x_Ge_y_Cu_z_Si_w_P_2_ exhibits a new X‐ray diffraction (XRD) pattern as shown in **Figure** **2**a, whose diffraction peaks are totally different from its elementary substances, revealing a new novel phase formation of such well‐designed HEM. More specially, when changing the feeding ratio from Zn_0.8_Ge_0.8_Cu_0.2_Si_0.2_P_2_ (HEM‐8822) to Zn_0.4_Ge_0.4_Cu_0.6_Si_0.6_P_2_ (HEM‐4466), those HEM analogues still keep the same XRD patterns without extra impurity peaks, suggesting a wide tunable solid solution range for those HEMs. When further increased the ratio to Zn_0.9_Ge_0.9_Cu_0.1_Si_0.1_P_2_ (HEM‐9911), the synthesized HEM‐9911 still keep the high purity phase (Figure S1a, Supporting Information, XRD measurement). However, when decreased the ratio to Zn_0.3_Ge_0.3_Cu_0.7_Si_0.7_P_2_ (HEM‐3377), there is an introduced impurity peak (Figure S1b, Supporting Information, CuP_2_ formation), revealing its solid solution limit floor. Therefore, those synthesized HEMs are verified to be one kind unique solid solution rather than simple metal‐phosphides, combining with a wide range tunable solid solution region and feed‐ratio floor of HEM‐4466. To further investigate the crystal structure and specific occupation of such HEMs, the XRD Rietveld refinements were performed in Figure 2b by using the GSAS software package. Rietveld results are shown in Figure 2c and Table S1 (Supporting Information), in which all of the error factors (e.g., wRp, Rp) are controlled within 0.06, revealing the high Rietveld quality. Accordingly, it is interestingly found such novel solid solutions can be well indexed to a new cubic system in F‐43m space group, 216. The refined cell parameters and crystal structures are identical to the ZnS structural analogue, in which the Zn, Si, Cu, and Ge share the equiatomic Zn site and the P atoms occupy the S sites. Besides, the refined atomic occupations (Table S1, Supporting Information) from HEM‐8822 to HEM‐4466 are consistent with their feeding ratios, suggesting the full reaction of their elementary substances after ball milling process. Among series HEM solid solutions, the Zn_0.5_Ge_0.5_Cu_0.5_Si_0.5_P_2_ (HEM‐5555) is found possessed the slightly largest cell parameters (e.g., *a* = 5.359 Å, *v* = 153.904 Å^3^), which may be attributed to its highest cation‐disorder degree. Though these HEM analogues possess different element proportion (Figure S2, Supporting Information, energy dispersion spectrum), they show similar morphology and comparable particle size. In Figure S3 (Supporting Information) of scanning electron microscope (SEM) measurement, typical Zn_x_Ge_y_Cu_z_Si_w_P_2_ particle contained of large number nanosized irregular primary stacks toward a micrometer level. To probe more accurate local information, the transmission electron microscopy (TEM) measurement was performed in Figure 1d. Typical HEM‐5555 primary particle is about 500 nm, in which the elemental Zn, Si, Cu, Ge, and P have uniformly distributed with each other at atomic level rather than simple mixing, without partial element aggregation. Further selected area electron diffraction (SAED) image in Figure S4a (Supporting Information) proves its cubic phase formation, and the high resolution transmission electron microscopy (HRTEM) image in Figure S4b (Supporting Information) also detected the d‐spacing of 0.19 nm matching well with its (220) phase. Besides, those HEM analogues possess high decomposition temperature over 550 °C (Figure S5, Supporting Information, thermogravimetric analysis), suggesting their relatively good structural stability. Consequently, a series of new cubic phase Zn_x_Ge_y_Cu_z_Si_w_P_2_ solid solutions with wide range tunable region are successfully synthesized.

### 2.3 Configurational Entropy Calculation and Characterization of Zn_x_Ge_y_Cu_z_Si_w_P_2_

**Figure 3:**
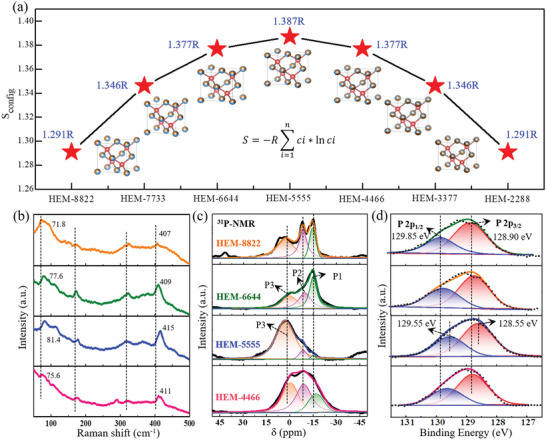
Configurational entropy characterization of Zn_x_Ge_y_Cu_z_Si_w_P_2_. Comparison of the calculated entropy values (a), Raman scattering (b), ^31^P‐NMR (nuclear magnetic resonance) spectra (c), and X‐ray photoelectron spectroscopy (XPS) profiles in P 2p region (d) for the Zn_x_Ge_y_Cu_z_Si_w_P_2_ solid solutions.

To further obtain the specific configurational entropy of above Zn_x_Ge_y_Cu_z_Si_w_P_2_ solid solutions, their entropies (*S*_config_) are calculated by the following equation:

(1) $$\left[S=-R\sum_{i=1}^{n}c_{i}\times \ln c_{i}\right]$$

where *c*_i_ represents the mole fractions of ions presented in the cation and anion site.^[^ ^31^ ^]^ As shown in **Figure** **3**a, according to above equation, the configurational entropy values of HEM‐8822, HEM‐6644, HEM‐5555, and HEM‐4466 are calculated to be 1.291R, 1.377R, 1.387R and 1.377R, respectively. Among them, it is interestingly found the HEM‐5555 accounts for the slightly largest configurational entropy due to its equiatomic occupation toward highest cation‐disorder degree. Such above entropy evolution was further detected by the Raman scattering, which is extrasensitive to local symmetry of materials as a local probe. In Figure 3b, all the HEM analogues deliver the typical Raman peaks at ≈71.8, ≈172.36, ≈322.25, and ≈407 cm^−1^, revealing their structural similarity. However, compared to HEM‐8822, the Raman peaks of other HEM gradually shift to higher values along with their increased configurational entropy. As proved, the HEM‐5555 shows the largest displacement (e.g., ≈81.4, ≈177.12, and 415 cm^−1^) among series HEM solid solutions (Figure S6, Supporting Information). To probe the intrinsic coordination environment in those solid solutions, the ^31^P solid‐state magic angle spinning nuclear magnetic resonance (MAS NMR) was performed in Figure 3c. Since the chemical shift is sensitive for local chemical environment, the ^31^P spectra can be decomposed into P1 (−14.95 ppm), P2 (−9.56 ppm), and P3 (0.15 ppm) signals for all of the HEM analogues, possibly corresponding to the tetrahedron, hexahedron, and octahedron condition, respectively.^[^ ^32^ ^]^ Interestingly, when changed the ratios from HEM‐8822 to HEM‐5555, the detected P1 and P2 peak signal gradually decreased while the P3 peak intensity become enhanced. When further changed the ratio from HEM‐5555 to HEM‐4466, the P1 and P2 peak signal increased back again while the P3 peak decreased. Such phenomena may be related to the structural evolution of above HEM counterparts, in which the HEM‐5555 possesses largest configurational entropy and highest cation‐disorder degree at equiatomic occupation, thus showing the largest P3 peak location. Similar conclusion can be made by the broadening of ^31^P spectra as well for the HEM‐5555. Furthermore, to investigate the surface composition and chemical states of above HEM solid solutions, the X‐ray photoelectron spectroscopy (XPS) was carried out in Figure S7 (Supporting Information), in which all of the elemental compositions including Si, Ge, Cu, Zn, and P can be well detected. For the high‐resolution P 2p peak in Figure 3d, it can be well deconvoluted into the typical P 2p_1/2_ and P 2p_3/2_ peaks for all HEM analogues, located at 129.85 and 128.90 eV, respectively. However, difference is that the P 2p_1/2_ and P 2p_3/2_ peaks of HEM‐5555 shift to lower binding energy (e.g., 129.55 and 128.55 eV), revealing its weak electron transfer of P element in HEM‐5555. Compared to other analogues, the larger entropy and higher cation‐disorder degree of HEM‐5555 result in its expandable cell parameters (Figure 1c) and thereby, triggering the weak metal‐P bonding interaction and lower P's electron transfer. Such weak metal‐P bonding interaction is considered to promote the reversibility and healable structure when served as the electrode material for LIBs.^[^ ^30^ ^]^

### 2.4 Electrochemical Performance Characterization

**Figure 4:**
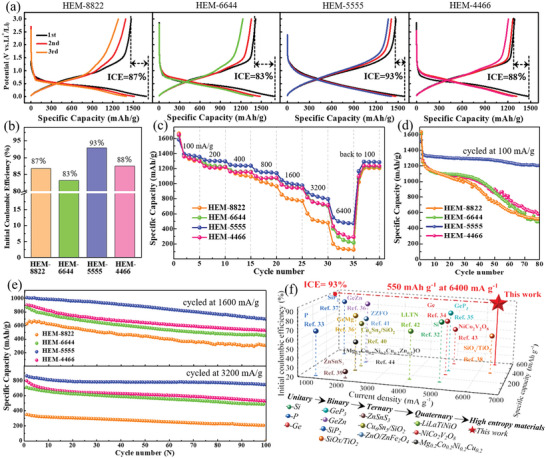
Electrochemical characterization of Zn_x_Ge_y_Cu_z_Si_w_P_2_ solid solutions. The discharge/charge profiles (a), initial coulombic efficiency (ICE) (b), rate capability (c), and cycle stability at low and high current density (d,e) of series Zn_x_Ge_y_Cu_z_Si_w_P_2_ electrodes. f) Comparison of the electrochemical performances of Zn_x_Ge_y_Cu_z_Si_w_P_2_ with published works.

When served as anode for LIBs, all of the well‐designed HEM solid solutions exhibit large capacity, suitable plateau, and high reversibility for advanced energy storage. As shown in **Figure** **4**a, the synthesized HEM‐8822, HEM‐6644, HEM‐5555, and HEM‐4466 electrodes, respectively, deliver 1694, 1703, 1581, and 1472 mAh g^−1^ for Li‐storage, combining with a low yet suitable lithiated platform of ≈0.5 V at the same time. Such large capacity output of series HEMs are benefitted from their rational component design, in which all of the Si, Ge, P, and Zn make contribution for Li‐storage by forming Li‐metal alloys. Therefore, this new finding on HEM solid solution design and synthesis has successfully introduced the high entropy concept into alloying type anode, breaking the conventional view that high entropy alloy is helpless for energy storage due to its transition metal composition. More specially, those well‐designed HEM solid solutions show relatively high reversibility for LIBs as well, delivering the ICE of 87% (HEM‐8822), 83% (HEM‐6644), 93% (HEM‐5555), and 88% (HEM‐4466), respectively, as shown in Figure 4b. Among them, the HEM‐5555 exhibits the highest ICE and best reversibility for LIBs, which may be ascribed to its largest configurational entropy and cation‐disorder degree. In Figure 4c and Figure S8 (Supporting Information), even conducted at a high current density of 6400 mA g^−1^ (about 4C‐rate), large reversible capacity of 201 mAh g^−1^ (15%, HEM‐8822), 403 mAh g^−1^ (30%, HEM‐6644), 551 mAh g^−1^ (40%, HEM‐5555), and 435 mAh g^−1^ (32%, HEM‐4466) can still be obtained, with small hysteresis (*ΔE*_p_) between its discharge and charge plateaus (Figure S9, Supporting Information), suggesting the superior fast charging ability and rate performances for those Zn_x_Ge_y_Cu_z_Si_w_P_2_ electrodes. Similarly, the HEM‐5555 exhibits the relatively best rate performances among series HEM analogues. In Figure 4d, when cycled at 100 mA g^−1^ for 80 cycles, large reversible capacity of 1204 mAh g^−1^ can still be obtained for the HEM‐5555 electrode, while other analogue electrodes suffer capacity fading, revealing the superior cyclability of HEM‐5555 as well. When cycled at 1600 mA g^−1^ even 3200 mA g^−1^ in Figure 4e and Figure S10 (Supporting Information) for long‐term cycling, such HEM analogues enable a superior cycle stability as well. Similarly, the HEM‐5555 electrode shows the relatively best cyclability, with remaining capacity of 747 mAh g^−1^ (at 1600 mA g^−1^) and 701 mAh g^−1^ (at 3200 mA g^−1^) after 100 cycles. To better evaluate the performance improvement of such configurational entropy evolution and certify its significance worked in metal‐phosphorus, we take the well‐designed HEM‐5555 products to compare with recent published work on anode materials. As shown in Figure 4f, when compared to unitary Si,^[^ ^33^ ^]^ P,^[^ ^34^ ^]^ Ge,^[^ ^35^ ^]^ binary GeP_3,_,^[^ ^36^ ^]^ GeMg,^[^ ^37^ ^]^ SiP_2_,^[^ ^38^ ^]^ SiO_x_/TiO_2_,^[^ ^39^ ^]^ ternary ZnSnS_3_,^[^ ^40^ ^]^ Cu_6_Sn_5_@SnO_2_,^[^ ^41^ ^]^ ZZFO,^[^ ^42^ ^]^ and quaternary LiLaTiNiO,^[^ ^43^ ^]^ NiCoV_2_O_8_,^[^ ^44^ ^]^ (Mg_0.2_Co_0.2_Ni_0.2_Cu_0.2_Zn_0.2_)O,^[^ ^45^ ^]^ and (FeMnNiCoCr)S_2_^[^ ^46^ ^]^, those well‐designed Zn_x_Ge_y_Cu_z_Si_w_P_2_ HEM solid solutions surpass all the above materials owing to its larger configurational entropy triggered higher reversibility (ICE = 93%) and excellent rate performances, greatly proving the efficiency and significance of high entropy concept in LIBs.

### 2.5 Volume Expansion Characterization

**Figure 5:**
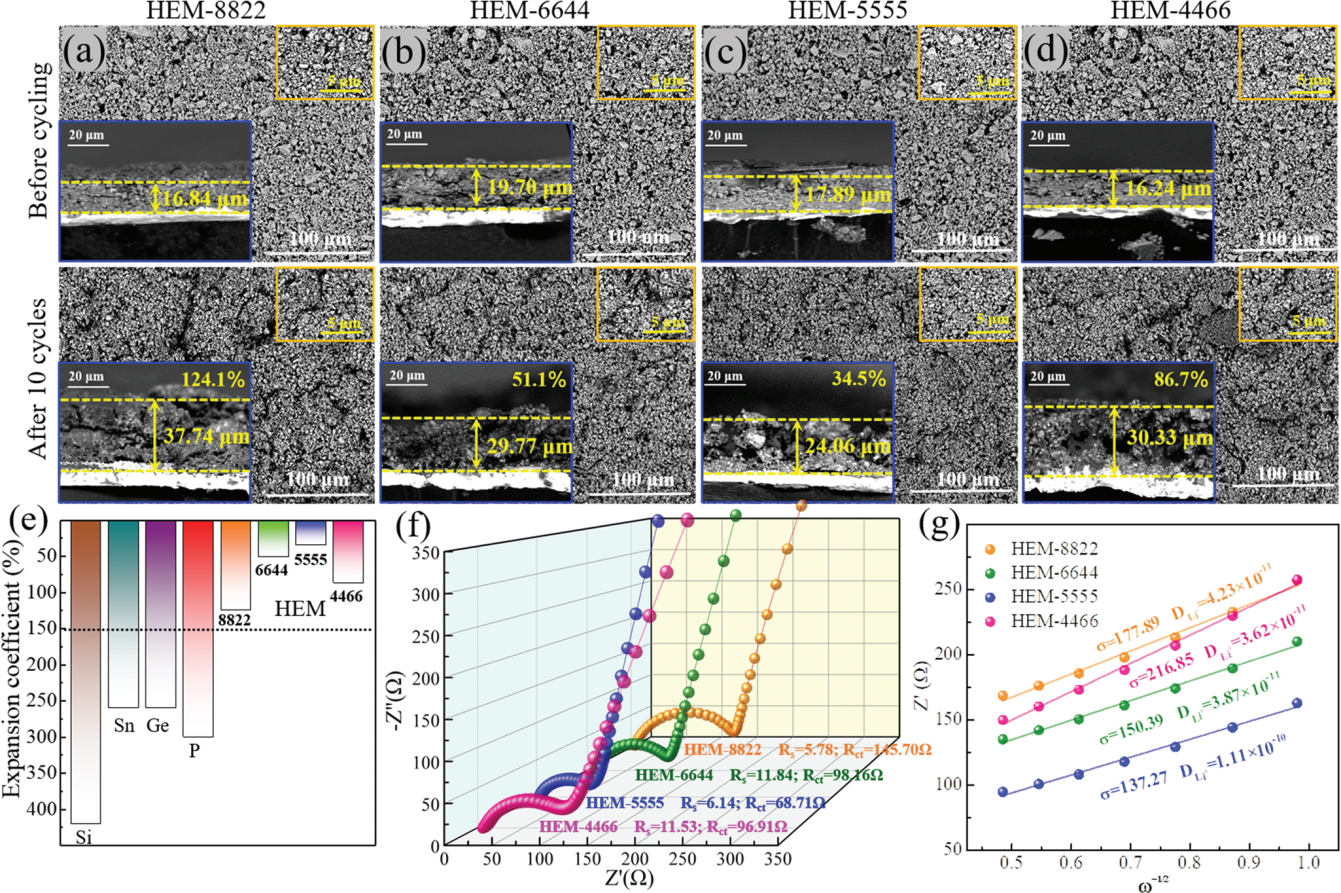
Volume expansion characterization of Zn_x_Ge_y_Cu_z_Si_w_P_2_. The morphologic scanning electron microscope (SEM) images and cross‐section observation before cycle and that after 10 cycles (a–d); comparison of the volume expansion coefficient (e), electrochemical impedance spectroscopy (EIS) measurement (f), and Li‐diffusivity (g) of series Zn_x_Ge_y_Cu_z_Si_w_P_2_ electrodes.

As well known, the volume expansion and particle pulverization of alloying anode material has always been an serious issue considering the cycle stability of batteries. To investigate the accommodation of volume changes after introducing high entropy concept, the morphology and cross‐section images before and after cycles for series HEM electrodes were performed in **Figure** **5**a–d. It can be seen that all of the above HEM solid solutions enable to keep the electrode integrity without obvious aggregation and pulverization after 10 cycles, combining with relatively low expansivity of 124.1% (HEM‐8822), 51.1% (HEM‐6644), 34.5% (HEM‐5555), and 86.7% (HEM‐4466), respectively. Obviously, the HEM‐5555 behaves the relatively best accommodation of volume changes owing to its largest configurational entropy and highest cation disorder degree. Nevertheless, compared to single elementary Si (420%), P (300%), Ge (260%), and Sn (260%) which suffer drastic volume expansion, series Zn_x_Ge_y_Cu_z_Si_w_P_2_ HEM solid solutions have greatly controlled the volume expansion within 150% after implementing the high entropy concept as shown in Figure 5e. Furthermore, the electrochemical impedance spectroscopy (EIS) measurements were also performed in Figure 5f. Generally, the intercept on the *x*‐axis refers the impedance of electrolyte (*R*_s_), and the following semicircles reflects the charge transfer resistance (*R*_ct_), and the remaining line in low‐frequency corresponds to the Li‐diffusion coefficient (*D*_Li+_).^[^ ^47^ ^]^ It can be seen that the fitting *R*_s_ and *R*_ct_ were calculated to be 5.78 and 145.70 Ω for HEM‐8822, 11.84 and 98.16 Ω for HEM‐6644, 6.14 and 68.71 Ω for HEM‐5555, and 11.53 and 96.91 Ω for HEM‐4466, respectively. Similarly, the HEM‐5555 behaves the comparable yet lowest charge transfer resistance. Besides, the *D*_Li+_ can also be calculated by the following equation^[^ ^48^ ^]^

(2) $$D_{\mathrm{Li}}=\frac{1}{2}{\left[\left(\frac{V_{m}}{FA\delta_{w}}\right)\frac{dE}{dx}\right]}^{2}$$

Where *V*_m_ refers the normalized molar volume of Zn_x_Ge_y_Cu_z_Si_w_P_2_, *F* is the Faraday constant (96 485 C mol^−1^), *A* is the contact area of electrode, *x* refers the charge transfer number of Zn_x_Ge_y_Cu_z_Si_w_P_2_, and *dE/dx* is the slope of discharge/charge profiles in Figure S11 (Supporting Information). *δ*_w_ refers the Warburg factor, which can be further obtained from the slope of *Z*’ versus *ω* ^−1/2^ line in Figure 5g:

(3) $$Z^{\prime }=R_{e}+R_{\mathrm{ct}}+\delta \omega^{-1/2}$$

where *ω* is the angular frequency in low frequency region. According to above equation, the Li‐diffusivity of HEM‐8822, HEM‐6644, HEM‐5555, and HEM‐4466 are calculated to be 4.23 × 10^−11^, 3.87 × 10^−11^, 1.11 × 10^−10^, and 3.62 × 10^−11^, respectively. Similarly, the HEM‐5555 exhibits the comparable yet slightly highest Li‐diffusivity and best reaction kinetics among series analogues, thus better reversibility and rate performance can be obtained.

### 2.6 Electrochemical Mechanism Characterization

**Figure 6:**
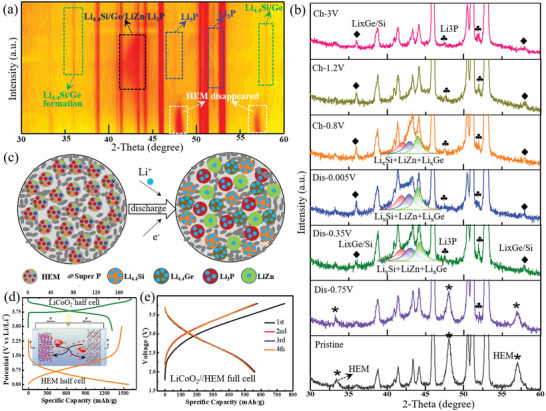
Electrochemical mechanism investigation for Zn_x_Ge_y_Cu_z_Si_w_P_2_. The in situ X‐ray diffraction (XRD) measurement (a,b) and corresponding reaction mechanism (c) of high‐entropy material 5555 (HEM‐5555) electrode. The discharge/charge profiles of HEM, LiCoO_2_ half cell (d) and assembled LiCoO_2_//HEM full battery (e).

Since the above Zn_x_Ge_y_Cu_z_Si_w_P_2_ is verified to be one kind unique solid solution rather than simple metal‐phosphide, the M‐P interactions (M = Zn, Ge, Cu, and Si) are not a strong covalent bond like metal‐phosphide and thereby, the conversion reaction process based on chemical bond breaking in metal phosphides would not take place in above Zn_x_Ge_y_Cu_z_Si_w_P_2_ solid solutions.^[^ ^30^, ^49^ ^]^ To investigate the Li‐reaction mechanism of above solid solution with large configurational entropy, taking HEM‐5555 as example, the in situ XRD measurement was carried out as shown in **Figure** **6**a,b. It can be seen that the typical diffraction peaks of HEM‐5555 gradually disappeared along with lithiation at the beginning. Subsequently, the diffraction peaks of Li_3_P, Li_x_Ge, Li_x_Si, and LiZn products slowly emerged in the following lithiated procedure. When fully discharged to 0.005 V, all of the lithiated Li_3_P, Li_x_Ge, Li_x_Si, and LiZn products can be well observed, suggesting the large capacity of HEM comes from its Li‐active Si, Ge, Zn, and P components. Accordingly, the following charging process greatly refers to the Li‐extraction of above Li‐metal alloys. Similarly, the cyclic voltammetry (CV) curves and d*Q*/d*V* profiles in Figure S12 (Supporting Information) also detected the lithiation and de‐lithiation of above Li‐metal behaviors. The corresponding electrochemical reaction mechanism of HEM solid solutions can be concluded in Figure 6c, in which the formative Li_3_P (≈0.7 V), Li_x_Ge (≈0.35 V), Li_x_Si (≈0.4 V), and LiZn (≈0.15 V) products occur at different but close plateaus, avoiding the integral overall volume expansion at a same time. Compared to its simple substances, there is a spatially confined and synergistic effect among the Si, Ge, Zn, and P components in such HEM solid solutions, conducting the Li_3_P, Li_x_Ge, Li_x_Si, and LiZn process in a more harmonious and gentle way. Besides, the high conductive Cu composition in HEM, though nonactive for Li‐storage, effectively bridges the fast electronic connection for the whole electrode. Consequently, those HEM analogues with high entropy stabilization enable a good accommodation of volume change and fast electron transportation to a certain degree, thus supporting the high reversibility, cyclability, and superior rate performances for LIBs. Finally, when assembled with commercial LiCoO_2_ cathode (Figure 6d), the formative LiCoO_2_//HEM full cell (Figure 6e) delivers a large reversible capacity of 562 mAh g^−1^, revealing the high potential of Zn_x_Ge_y_Cu_z_Si_w_P_2_ as an attractive high entropy anode for advanced energy storage.

## 3 Conclusion

In summary, to promise the HEM with Li‐activity and introduce the high configurational entropy into alloying type anode, traditional HEM is well redesigned using the Li‐active Si, Ge, P, and Zn elements to replace the nonactive transition metal components. Interestingly, one kind novel Zn_x_Ge_y_Cu_z_Si_w_P_2_ solid solutions with Li‐activity are successfully synthesized as a proof of concept. Such formed Zn_x_Ge_y_Cu_z_Si_w_P_2_ analogues possess a new structure phase, which is first verified to belong to the cubic system in F‐43m space group, combining with a wide range tunable solid solution range (from 9911 to 4466) as well. Among them, the HEM‐5555 accounts for the largest configurational entropy and highest cation‐disorder degree. Various experimental tools and theoretical calculations are performed to investigate the structural and physicochemical properties of those solid solutions. Benefitted from above rational design strategy, such synthesized Zn_x_Ge_y_Cu_z_Si_w_P_2_ deliver large capacity (>1500 mAh g^−1^), suitable plateau (≈0.5 V) and high reversibility for LIBs, breaking the conventional view that high entropy alloy is helpless for energy storage due to its transition metal composition. Among them, the target Zn_0.5_Ge_0.5_Cu_0.5_Si_0.5_P_2_ product exhibits the comparable yet highest ICE (93%), Li diffusion coefficient (1.11 × 10^−10^), lowest volume expansion (34.5%), and more specially, the best rate performances (551 mAh g^−1^ at 6400 mA g^−1^) owing to its highest configurational entropy. Possible mechanism reveals that the high entropy stabilization helps to buffer the volume change and facilitates electronic transportation to a large extent, thus supporting the reversibility, cyclability, and better rate performances. This new finding on design and synthesis of Zn_x_Ge_y_Cu_z_Si_w_P_2_ greatly mitigates the industry's reliance on single critical metal source and inject fresh blood into alloying anode family for fast charging LIBs. And the implement of high configurational entropy into metal‐phosphorus solid solution may open an avenue to material design and electrochemical optimization toward advanced energy storage.

## 4 Experimental Section

### Material Synthesis

A series of the high entropy metal‐phosphorus solid solutions were synthesized by a typical high energy mechanical ball milling method. Take the HEM‐5555 as example, elementary Zn, Ge, Cu, Si, and P powders in molar ratio of 0.5:0.5:0.5:0.5:2 were fully mixed and then transferred into a stainless steel jar under Ar atmosphere (assembled in a glove box). After continuously ball milling for 15 h with a rotational speed of 400 rpm min^−1^, the HEM‐5555 product with high purity can be successfully obtained. Similarly, other HEM‐8822, HEM‐6644, and HEM‐4466 analogues in molar ratios of 0.8:0.8:0.2:0.2:2, 0.6:0.6:0.4:0.4:2, 0.4:0.4:0.6:0.6:2 can also be synthesized by following above procedures.

### Material Characterization

The synthesized high entropy metal‐phosphorus was well characterized by XRD (Bruker AXS D2 PHASER), TEM (JEOL JEM 2100, Japan), Raman spectrometer (Thermo fisher Scientific DXRxi with 532 nm excitation laser, USA), NMR (Bruker, 1.4T, 600.21 MHz), XPS (Axis Supra), and thermogravimetric analysis (TGA, PerkinElmer). The morphology features were performed by the scanning electron microscopy and energy dispersion spectrum (Phenom ProX, Phenom‐World BV, Eindhoven, Netherlands), and the crystal structures were refined by a Rietveld method using GSAS software package.

### Electrochemical Characterization

To obtain the electrodes, the HEM active materials were firstly mixed with conductive carbon (Super P) and LiOH–polyacrylic acid binder (Li‐PAA) in a mass ratio of 7:2:1. Then, the obtained sizing agent was coated onto a copper foil following a drying procedure (80 °C for 12 h in vacuum box). Finally, the electrodes with diameter of 10 mm can be cut out with a slicer. The electrodes were calended pressures when applicable. The mass loading of electrode material was about ≈2.5 mg cm^−2^. A total of 2032 coin‐type batteries were assembled in a glove box (H_2_O, O_2_ <1 ppm) by using above HEM materials as working electrodes and lithium metal (diameter of 10 mm, thickness of 0.55 mm) as counter electrode. The electrolyte in this experiment was the 1.0 mol L^−1^ LiPF_6_ dissolved in ethylene carbonate (EC), dimethyl carbonate (DMC), and ethyl methyl carbonate (EMC) (EC:DMC:EMC = 1:1:1 vol%) solution. Galvanostatic discharge/charge profiles between 0.005 and 3.0 V, CV and high rate performances at different current rate were performed on the Neware battery test system (CT‐4008T‐5V10mA‐164, 25 °C) and the Bio‐Logic, EC‐lab (VSP‐300, France). The EIS measurement was carried out on a high‐precision electrochemical impedance testing system (Solartron 1260 + 1470E). And the d*Q*/d*V* curves can be obtained by using the Hokuto Denko automatic battery testing system (HJ1001SD8, Gifu, Japan).

## Conflict of Interest

The authors declare no conflict of interest.

## Supporting information

Supporting Information

## Data Availability

The data that support the findings of this study are available in the supplementary material of this article.
