# Supplementary material for: Understanding the Configurational Entropy Evolution in Metal‐Phosphorus Solid Solution for Highly Reversible Li‐Ion Batteries
Source: Adv Sci (Weinh). 2023 Feb 15;10(9):2300271. doi: 10.1002/advs.202300271 (PMC10037993; doi:10.1002/advs.202300271)
Supplement: Supplementary file 1 — Supporting Information [file ADVS-10-2300271-s001.pdf]

ADVANCED SCIENCE
Open Access

Supporting Information

for *Adv. Sci.*, DOI 10.1002/advs.202300271

Understanding the Configurational Entropy Evolution in Metal-Phosphorus Solid Solution for Highly Reversible Li-Ion Batteries

Yaqing Wei, Runzhe Yao, Xuhao Liu, Wen Chen, Jiayao Qian, Yiyi Yin, De Li* and Yong Chen**

Supporting Information

Understanding the configurational entropy evolution in metal-phosphorus solid solution for highly reversible Li-ion batteries

Yaqing Wei [a,#,*], *Runzhe Yao* [a,#], *Xuhao Liu* [a,#], *Wen Chen* [a], *Jiayao Qian* [a], *Yiyi Yin* [a], *De Li* [a,*] *and Yong Chen* [b,*]

a Prof. Y. Q. Wei, R. Z. Yao, X. H. Liu, W. Chen, J. Y. Qian, Y. Y. Yin, Prof. D. Li
State Key Laboratory of Marine Resource Utilization in South China Sea, Hainan Provincial Key Laboratory of Research on Utilization of Si-Zr-Ti Resources, School of Materials Science and Engineering, Hainan University, 58 Renmin Road, Haikou 570228, Hainan, P. R. China

b Prof. Y. Chen
Guangdong Key Laboratory for Hydrogen Energy Technologies; School of Materials Science and Hydrogen Energy, Foshan University, 528000, Foshan, P. R. China

# These authors contributed equally to this work.

* Corresponding author E-mail:
yqwei@hainanu.edu.cn; lidenju@sina.com; ychen2002@163.com

Keywords: high entropy alloy, anode material, volume expansion, initial coulombic efficiency, lithium-ion batteries

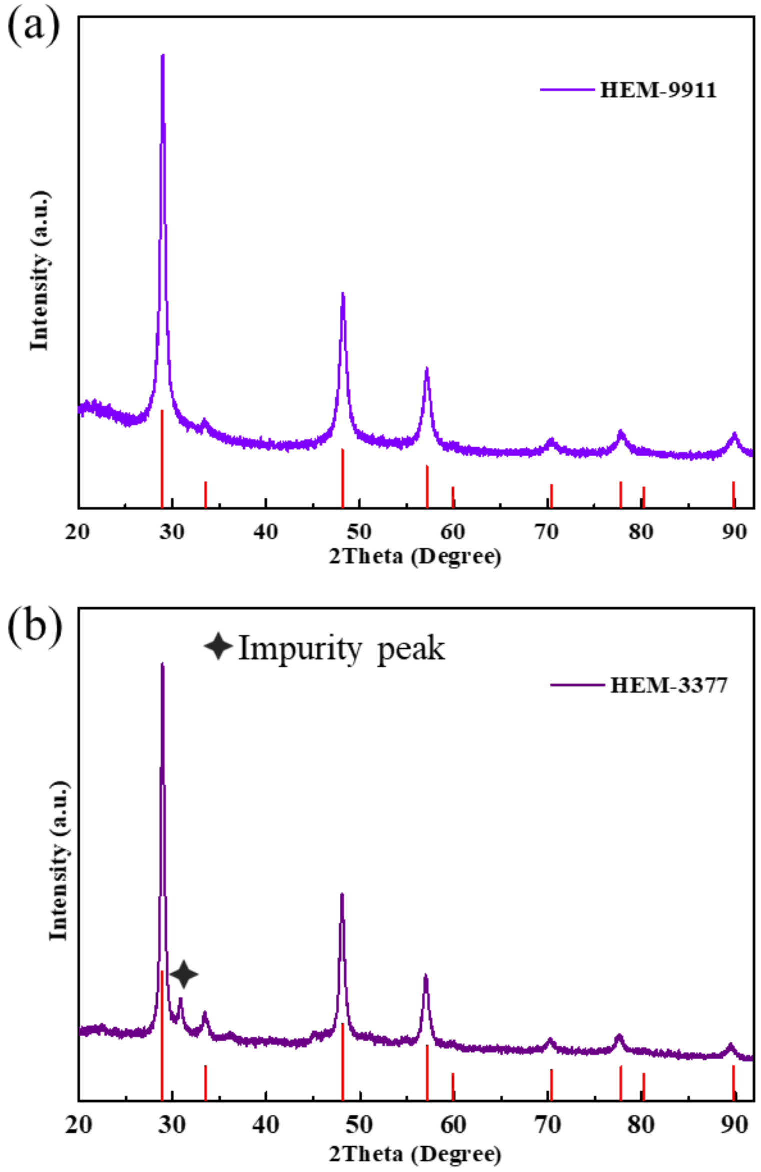

Figure S1. The XRD patterns of synthesized HEM-9911 (a) and HEM-3377 (b) powders.

Table S1. The fractional atomic coordinates, elementray occupation and isotropic displacement parameters (Å^2) of series $Zn_xGe_yCu_zSi_wP_2$ HEM materials.

		x	y	z	Occ.
HEM-8822	Zn	0	0	0	0.3894
	Si	0	0	0	0.3827
	Ge	0	0	0	0.1173
	Cu	0	0	0	0.1106
	P	0.25	0.25	0.25	1.0000
HEM-6644	Zn	0	0	0	0.3012
	Si	0	0	0	0.2976
	Ge	0	0	0	0.2024
	Cu	0	0	0	0.1988
	P	0.25	0.25	0.25	1.0000
HEM-5555	Zn	0	0	0	0.2493
	Si	0	0	0	0.2502
	Ge	0	0	0	0.2498
	Cu	0	0	0	0.2507
	P	0.25	0.25	0.25	1.0000
HEM-4466	Zn	0	0	0	0.1988
	Si	0	0	0	0.1878
	Ge	0	0	0	0.3122
	Cu	0	0	0	0.3012
	P	0.25	0.25	0.25	1.0000

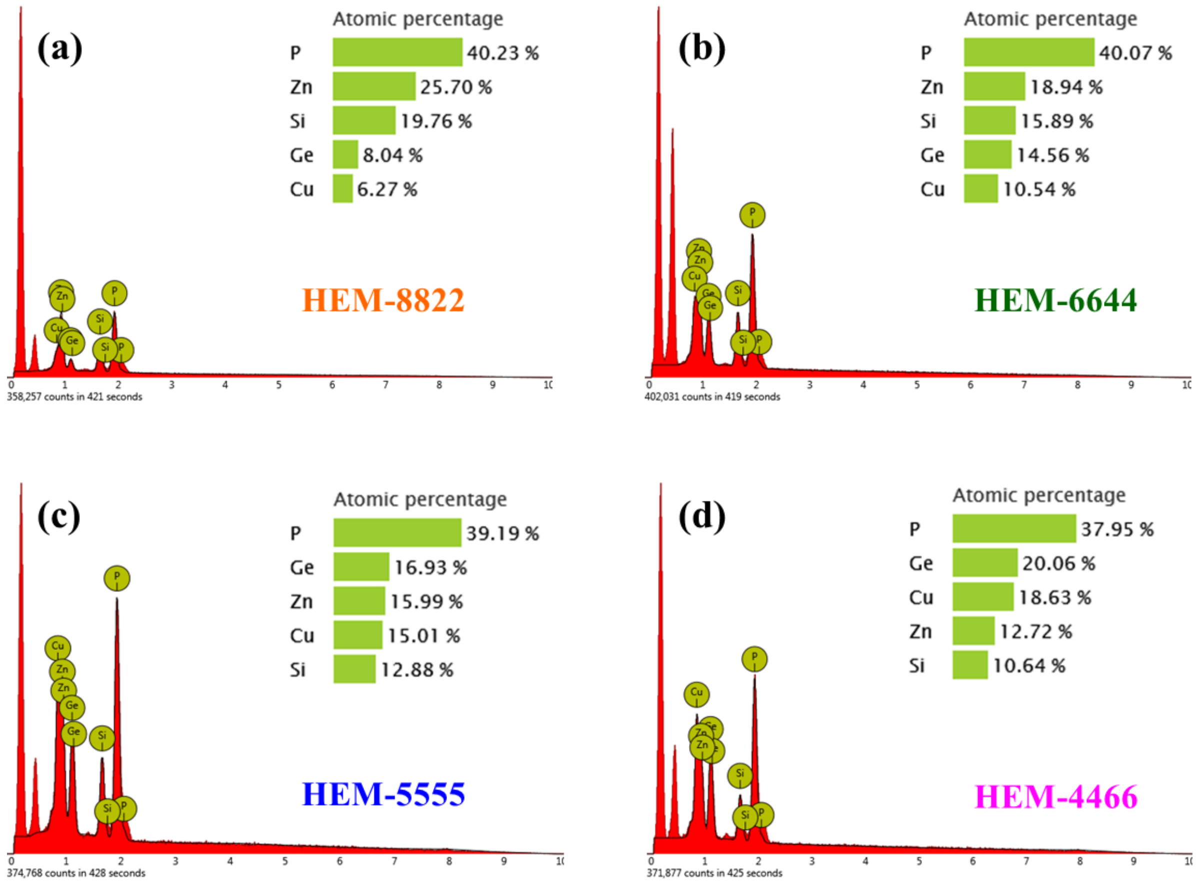

Figure S2. The elementray energy dispersion spectrum (EDS) of synthesized HEM-8822 (a), HEM-6644 (b), HEM-5555 (c) and HEM-4466 (d) analogues.

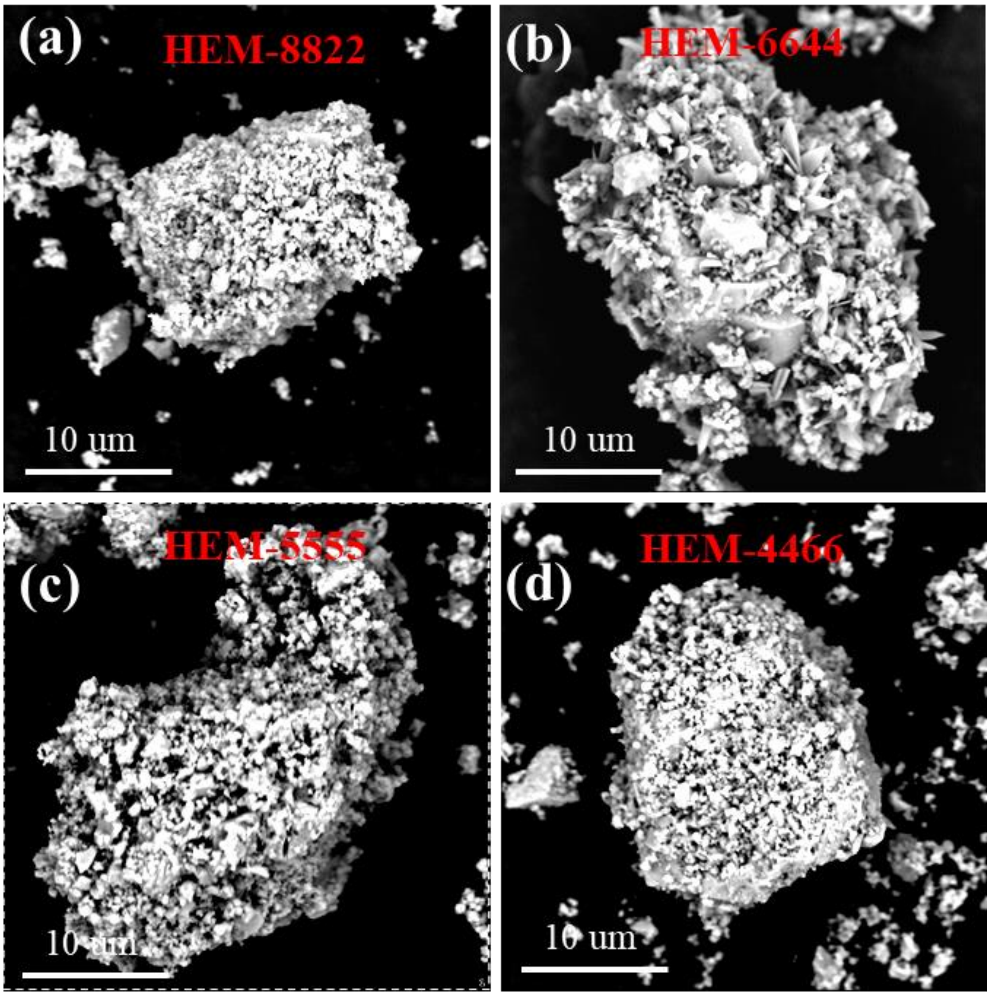

Figure S3. The SEM images of synthesized HEM-8822 (a), HEM-6644 (b), HEM-5555 (c) and HEM-4466 (d) analogues.

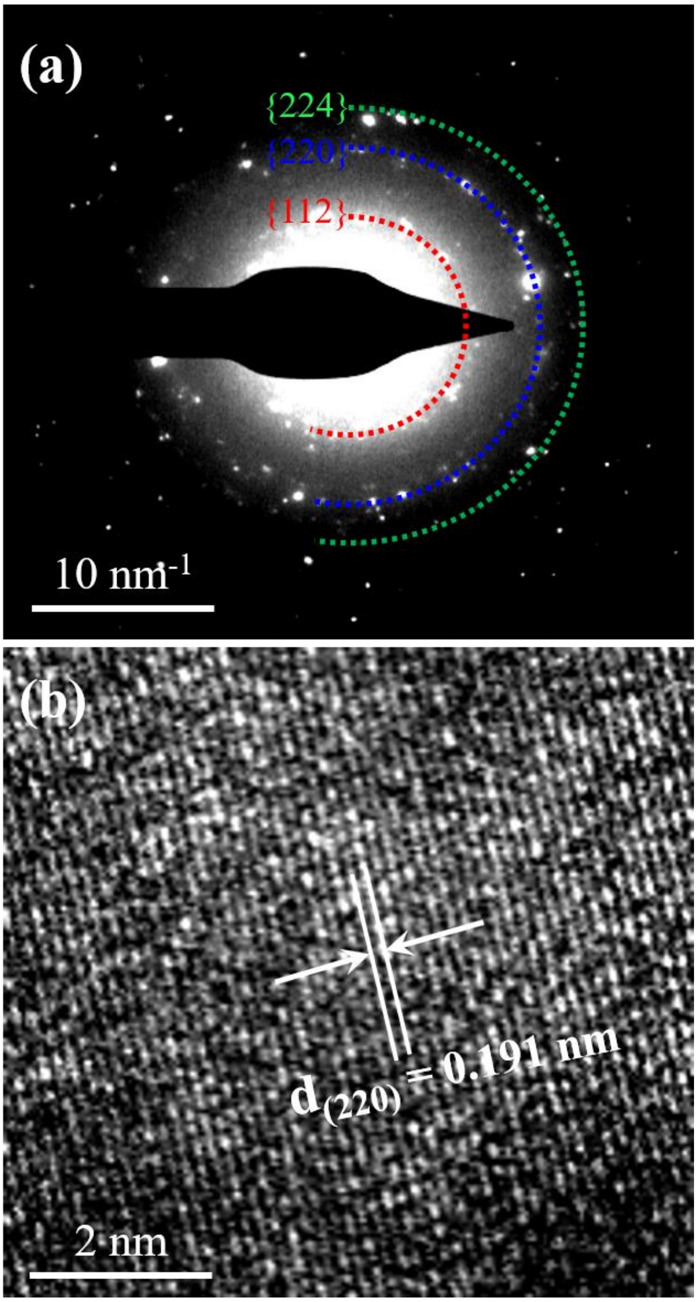

Figure S4. The SAED image (a) and HRTEM image (b) of typical HEM-5555 particle.

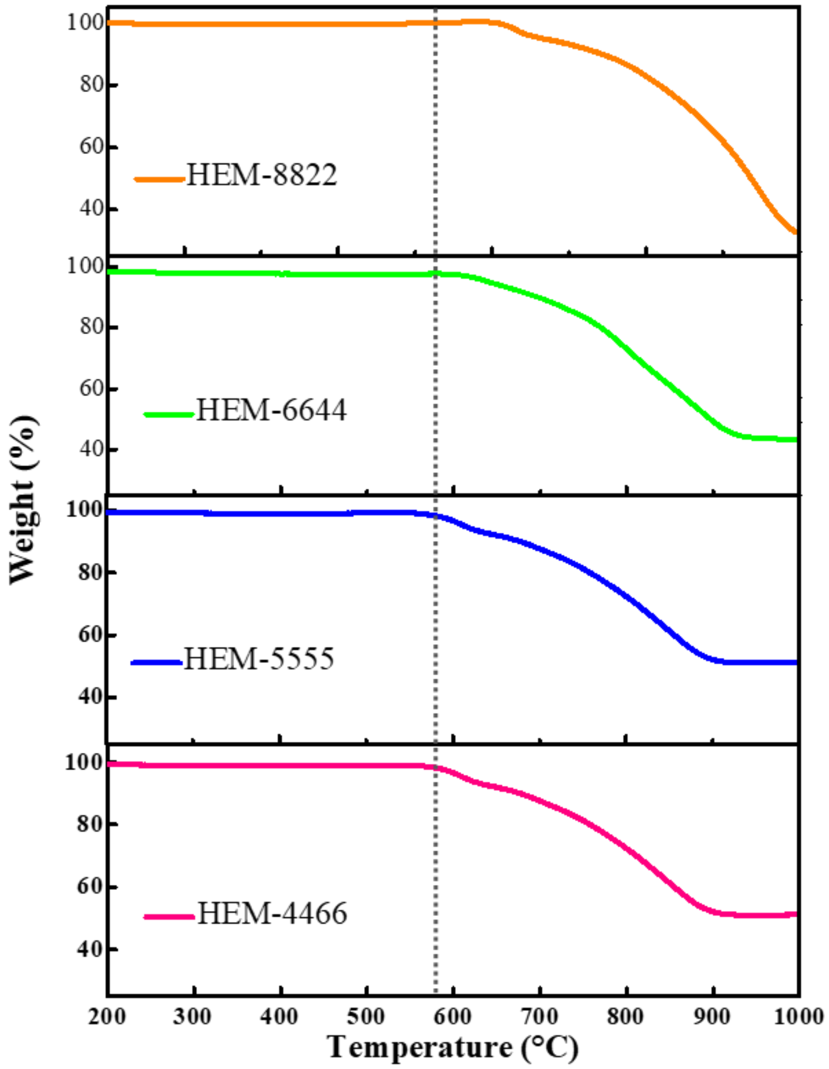

Figure S5. Thermogravimetric analysis of series $Zn_xGe_yCu_zSi_wP_2$ HEM materials.

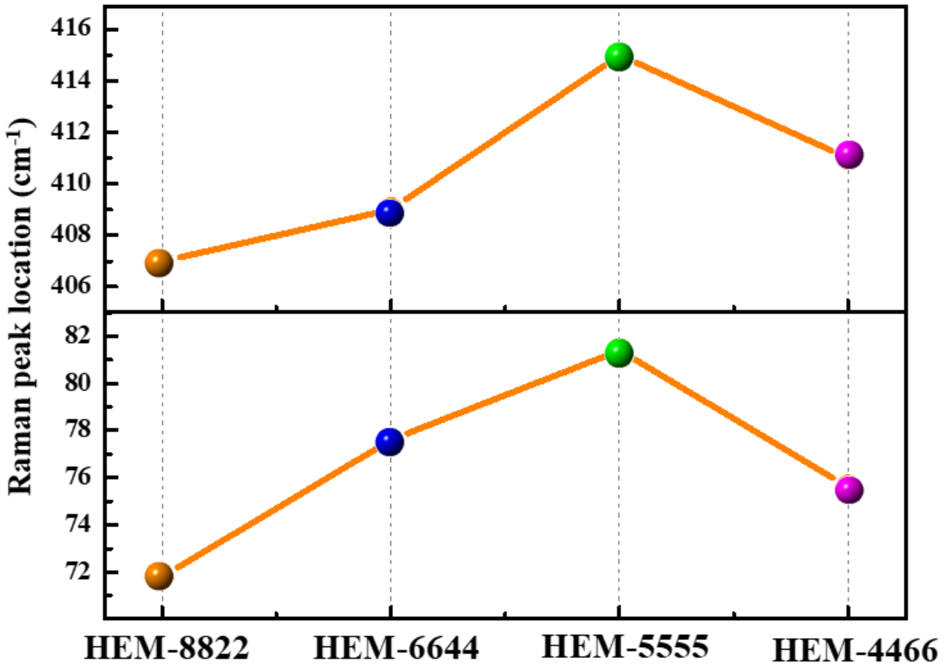

Figure S6. Comparison of the Raman peak location between series $Zn_xGe_yCu_zSi_wP_2$ analogues, in which the HEM-5555 delivers the largest disaplacement of peak location owing to its highest cation disorder degree and configurational entropy.

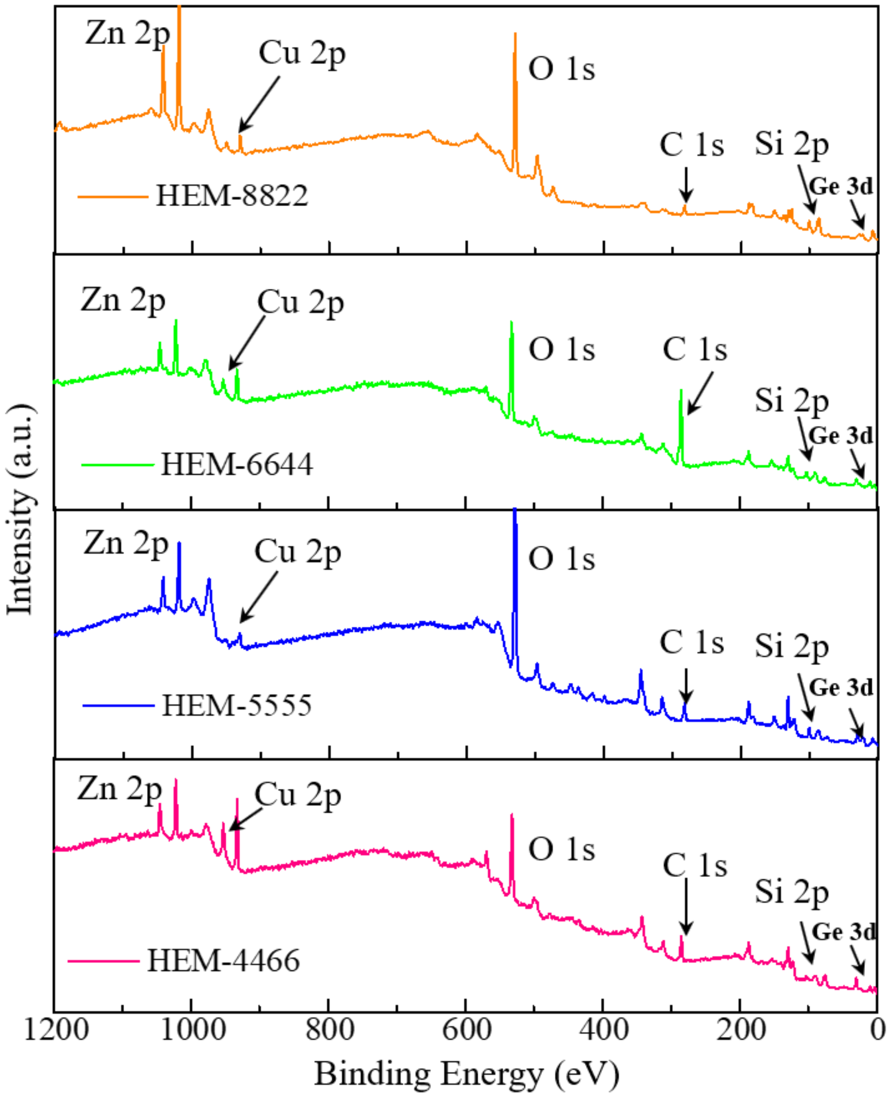

Figure S7. The XPS full spectra of series $Zn_xGe_yCu_zSi_wP_2$ HEM analogues.

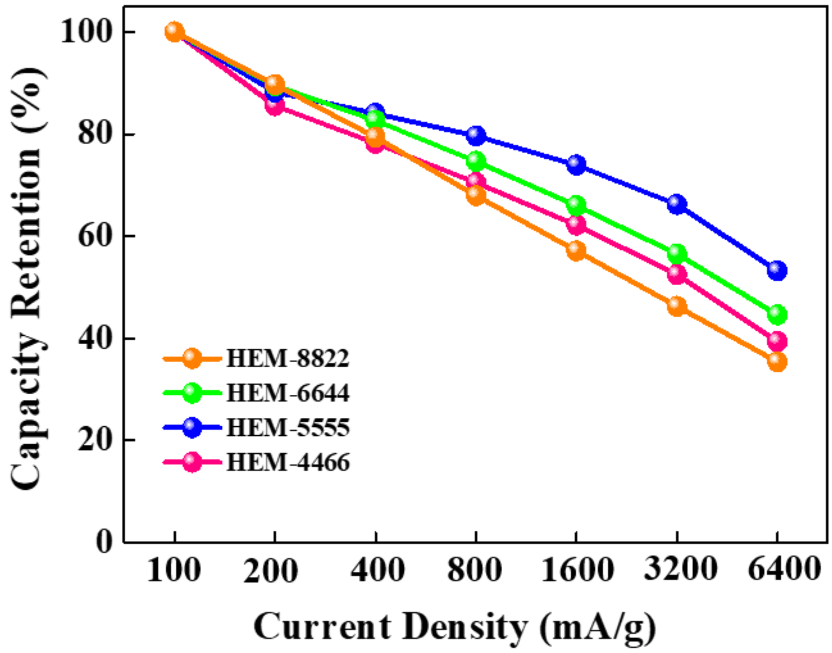

Figure S8. The capacity retention at various current densities of $Zn_xGe_yCu_zSi_wP_2$ electrodes.

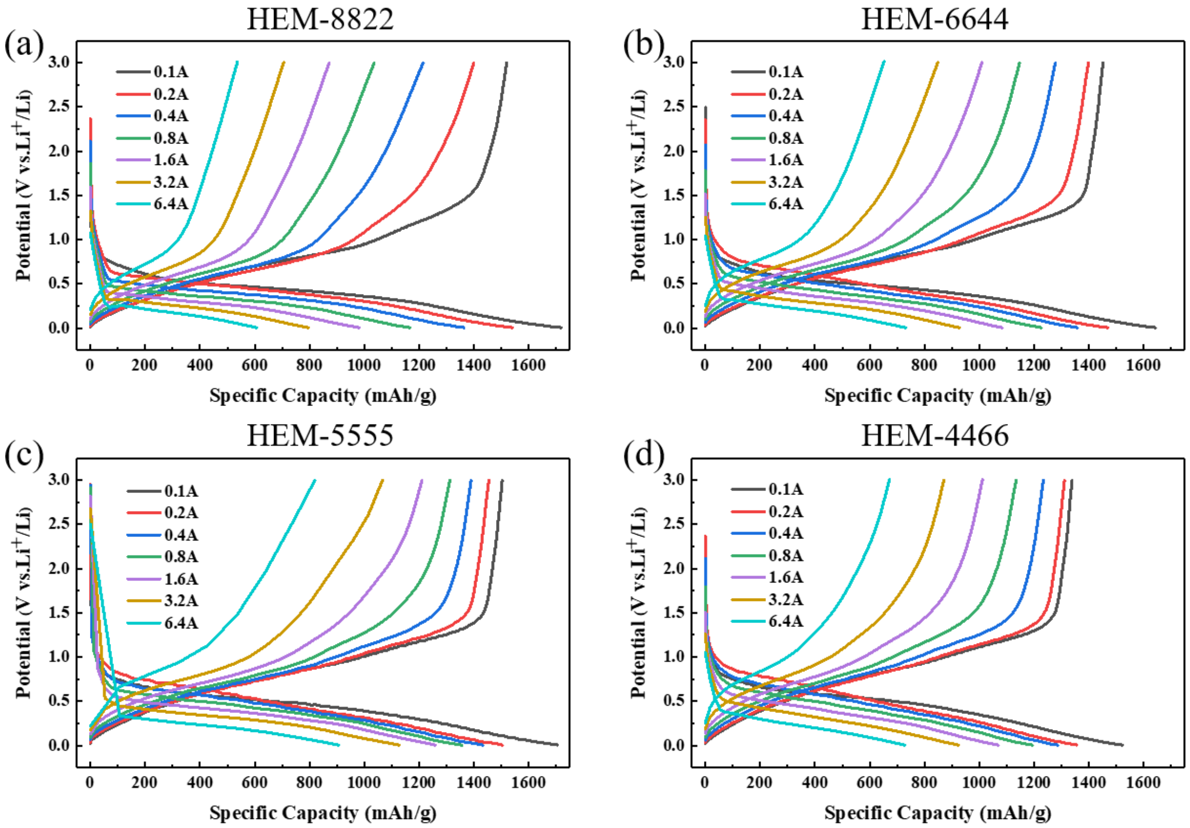

Figure S9. The discharge/charge profiles at various current densities of HEM-8822 (a), HEM-6644 (b), HEM-5555 (c) and HEM-4466 (d) electrodes.

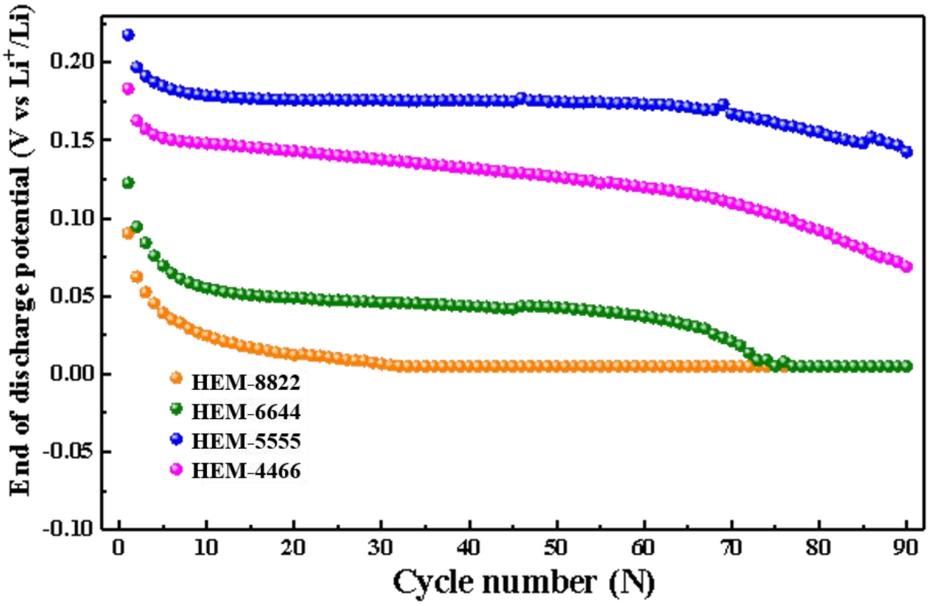

Figure S10. The end of discharge potential versus cycle number when operated with a limited capacity of 1000 mAh/g for series $Zn_xGe_yCu_zSi_wP_2$ electrodes.

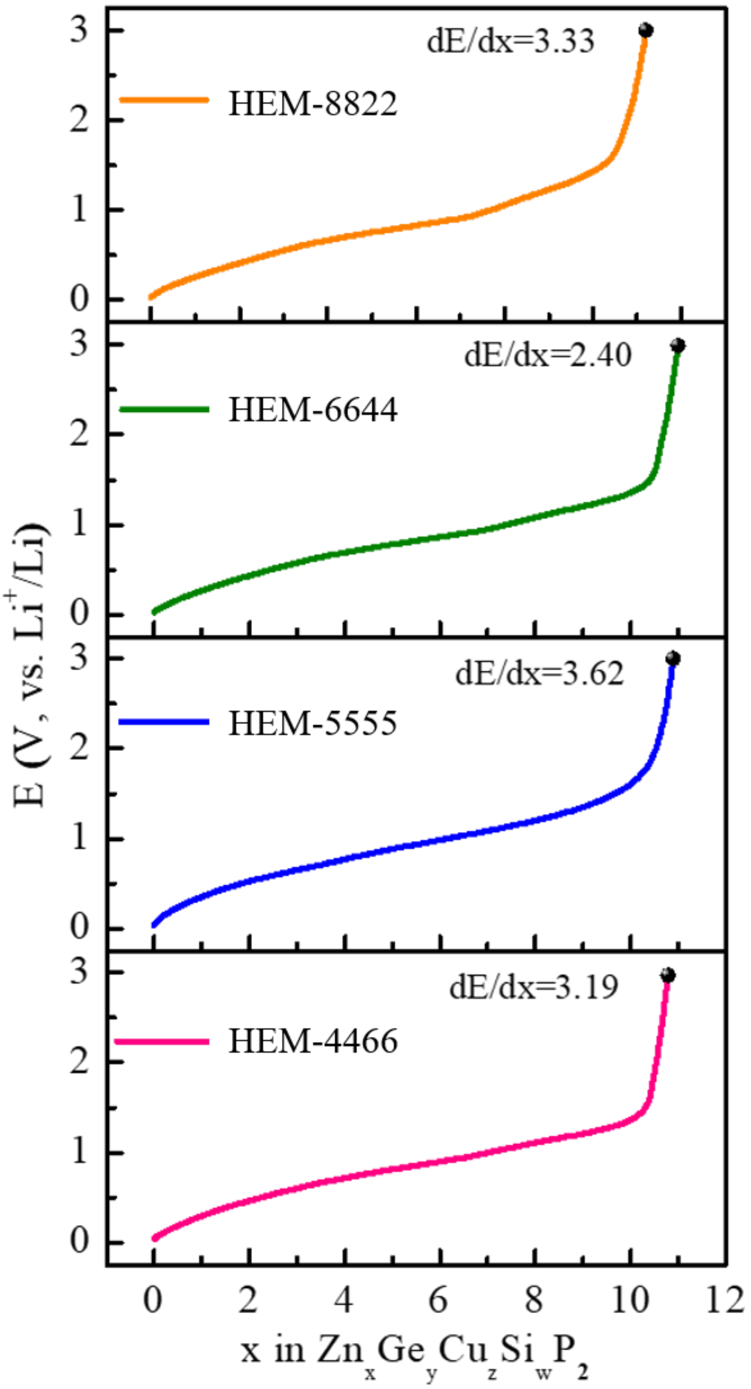

Figure S11. The discharge potential *E* versus *x* in $Zn_xGe_yCu_zSi_wP_2$ electrodes.

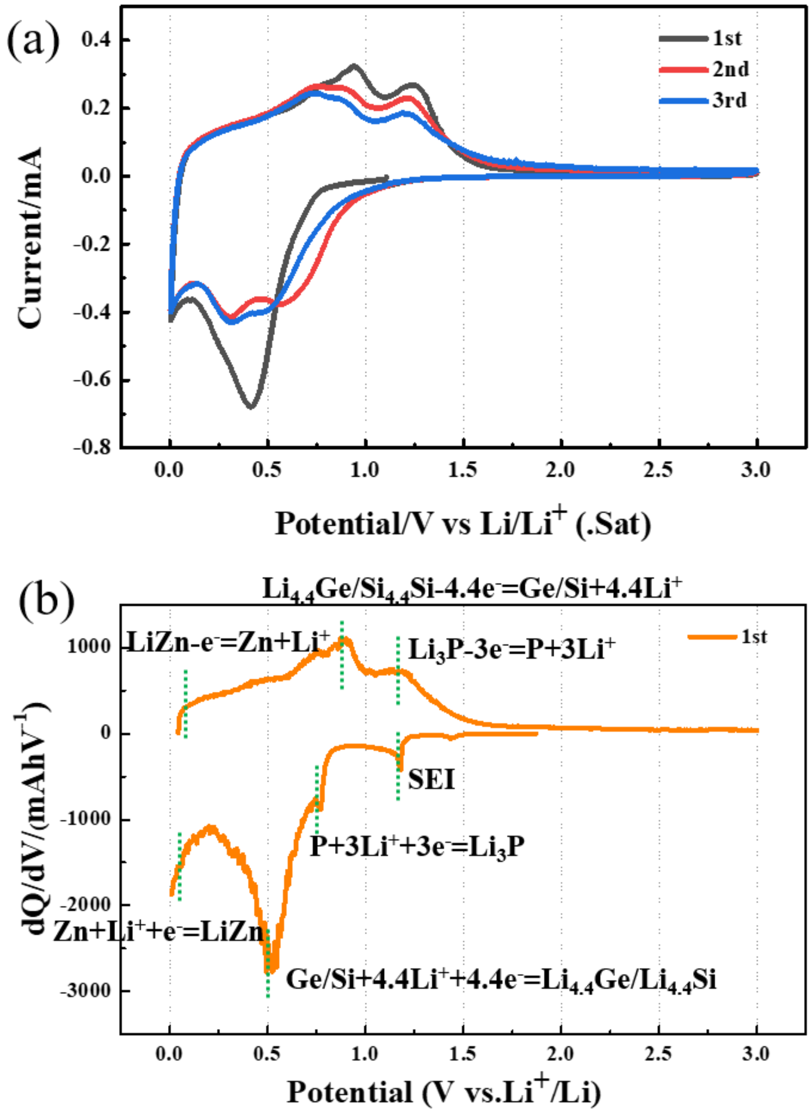

Figure S12. The cyclic voltammetry curves (a) and dQ/dV profiles (b) of HEM-5555 electrode.
